# Therapeutic advances in eculizumab for atypical hemolytic uremic syndrome: a narrative review

**DOI:** 10.3389/fphar.2026.1834141

**Published:** 2026-05-28

**Authors:** Dandan Zhang, Yichen Xiao, Hualin Ma

**Affiliations:** 1 Department of Nephrology, Shenzhen People’s Hospital, The Second Clinical Medical College, Jinan University, Shenzhen, China; 2 Department of Nephrology, The First Affiliated Hospital, School of Medicine, Southern University of Science and Technology, Shenzhen, China; 3 Department of Nephrology, Shenzhen People’s Hospital (The Second Clinical Medical College, Jinan University, The First Affiliated Hospital, Southern University of Science and Technology), Shenzhen, Guangdong, China; 4 Shenzhen Key Laboratory of Kidney Diseases, Shenzhen, Guangdong, China; 5 Shenzhen Clinical Research Center for Geriatrics, Shenzhen People’s Hospital, Shenzhen, Guangdong, China

**Keywords:** atypical hemolytic uremic syndrome, complement inhibitor, dose individualization, eculizumab, thrombotic microangiopathy, treatment discontinuation

## Abstract

Atypical hemolytic uremic syndrome (aHUS) represents an uncommon, life-threatening complement-mediated thrombotic microangiopathy that is linked to both significant renal and extrarenal complications. Eculizumab, a monoclonal antibody targeting complement component C5, has dramatically improved patient outcomes and is widely recognized as a first-line therapy. However, uncertainty remains regarding the optimal dosing regimen, therapeutic monitoring, feasibility of treatment discontinuation, and appropriate duration of therapy in patients with aHUS. This narrative review is based on a PubMed literature search conducted through 2025, with search terms encompassing aHUS, eculizumab, complement inhibitor, treatment discontinuation, and dosing strategies. By integrating evidence from key clinical trials, observational cohorts, and registry-based analyses, a comprehensive evaluation of eculizumab’s efficacy and safety profiles is presented. We discuss individualized management strategies, with particular emphasis on dose optimization. Importantly, we focus on the available evidence regarding eculizumab discontinuation, addressing its feasibility, relapse risk, and post-discontinuation monitoring strategies, and suggest that cautious treatment withdrawal can be considered in selected patients under close surveillance. In addition, tailored management approaches for specific aHUS subtypes are discussed. In conclusion, we outline future directions for complement C5 inhibition, emphasizing that the advancement and clinical implementation of novel C5 inhibitors may offer new opportunities for the long-term management of aHUS.

## 1 Introduction

Atypical hemolytic uremic syndrome (aHUS) represents a complement-driven microangiopathy (TMA) distinguished by the classic triad of microangiopathic hemolytic anemia (MAHA), thrombocytopenia, and acute kidney injury (Noris and Remuzzi, 2009; Fakhouri et al., 2017a; Walsh and Kavanagh, 2025). It is rare, with an estimated annual incidence of 0.23–1.9 per million people (Yan et al., 2020), and appears to be slightly more common in children than in adults (Mele et al., 2014). The underlying pathophysiology is dysregulated activation of the alternative complement pathway, most often owing to inherited or acquired defects in complement regulation. Pathogenic variants have been described in genes encoding complement factor H (CFH), complement factor I (CFI), membrane cofactor protein (MCP/CD46), C3 and complement factor B (CFB). The most frequently acquired abnormality is anti–factor H autoantibodies (FHAA) (Walsh and Kavanagh, 2025). Additional sporadic etiologies of aHUS include infection, pregnancy, drug exposure, and organ transplantation (Noris and Remuzzi, 2009). Unregulated complement activation leads to excessive generation of C5 convertase and formation of the membrane attack complex (MAC), resulting in endothelial damage and microvascular thrombosis (Fakhouri et al., 2017b; Raina et al., 2019). In addition to predominant renal injury, aHUS can lead to severe complications such as ischemic cerebrovascular events and atherosclerosis (Noris and Remuzzi, 2009; Sokola et al., 2023). Before complement inhibition, outcomes were poor, with reported mortality rates of up to 25% and progression to end-stage kidney disease in approximately half of patients (Noris and Remuzzi, 2009; Fremeaux-Bacchi et al., 2013).

Currently, aHUS continues to be a diagnosis of exclusion due to the absence of a definitive confirmatory test (Fakhouri et al., 2023). In clinical practice, evaluating a patient with TMA necessitates the systematic exclusion of alternative causes of hemolytic uremic syndrome, such as infection caused by Shiga toxin-producing *Escherichia coli* (STEC) or *Streptococcus pneumoniae*. Thrombotic thrombocytopenic purpura (TTP) should also be ruled out, typically by severely reduced ADAMTS13 activity and/or an inhibitor (Raina et al., 2019). Although testing for complement protein levels and screening for genetic variants are not standalone diagnostic criteria, they support the diagnosis, prognostic evaluation, and monitoring of treatment response (Multidisciplinary Consensus Collaboration Group on Atypical Hemolytic Uremic Syndrome, 2025). When the diagnosis is uncertain, renal biopsy can offer crucial pathological evidence (Goodship et al., 2017). Uncommon inherited disorders, including cobalamin C deficiency and diacylglycerol kinase ε (DGKE) deficiency can resemble aHUS but involve distinct mechanisms and necessitate alternative treatments (Fakhouri et al., 2017a).

Plasma exchange (PE) and plasma infusion (PI) represent the conventional treatments for aHUS, with the aim of removing circulating autoantibodies and replacing deficient complement regulators. However, plasma therapy essentially remains a supportive modality that fails to address the underlying dysregulation of the complement cascade (Waters and Licht, 2011). Furthermore, its efficacy exhibits considerable interindividual variability, particularly depending on the specific genetic mutation involved. Treatment is also linked to elevated relapse rates upon cessation and, when required for the long term, entails risks such as catheter-related infections and thrombosis (Raina et al., 2019; Wijnsma et al., 2019a).

The recognition of complement dysregulation as the central mechanism in aHUS established complement-targeted therapy as a novel therapeutic direction. Eculizumab, a humanized monoclonal antibody, binds to C5 and inhibits its cleavage by C5 convertase into C5a and C5b. This action prevents the formation of the terminal MAC, thereby intercepting the downstream cascade of endothelial damage and thrombus formation (Legendre et al., 2013). Following its initial approval for aHUS in 2011, the efficacy and safety of eculizumab were confirmed in two prospective, open-label, pivotal phase II trials (Legendre et al., 2013; Licht et al., 2015), supporting its subsequent integration into clinical practice as a first-line treatment.

This review synthesizes current evidence on eculizumab in patients with aHUS, underscoring its transformative role in improving clinical outcomes. We critically evaluate its efficacy, safety profile, and evolving strategies for dose individualization. A primary focus is the feasibility of treatment cessation, incorporating recent recommendations on therapy duration, criteria for cessation, and post-cessation monitoring protocols. Its application in high-risk subgroups has also been discussed. Finally, we contextualize the position of eculizumab within the growing spectrum of complement inhibitors, comparing it with the longer-acting agent ravulizumab.

## 2 Efficacy of eculizumab

Multiple prospective clinical studies and real-world evidence collectively demonstrate the significant clinical efficacy of eculizumab in patients with aHUS (Supplementary Table S1).

In two prospective, open-label phase II trials, Trial 1 enrolled 17 patients with acute, progressive TMA, with changes in platelet count designated as the primary measure to address the urgent need to halt complement-driven thrombocytopenia in rapidly deteriorating clinical conditions. In this trial, eculizumab treatment led to a significant increase in platelet count within the first week. In the overall population, 82% of the patients had a normal platelet counts at week 26. The estimated glomerular filtration rate (eGFR) increased by 32 mL/min/1.73 m^2^ at week 26, and 80% of patients were able to discontinue dialysis (Legendre et al., 2013). In contrast, Trial 2 involved 20 patients in the chronic, stable phase on maintenance plasma therapy. The primary endpoint for this trial was centered on TMA event-free status, designed to reflect long-term stability rather than acute improvement. By week 26, 80% of patients achieved this endpoint, though the mean improvement in eGFR was modest (6 mL/min/1.73 m^2^), indicating that earlier intervention may lead to more significant improvement (Legendre et al., 2013). Long-term extension data showed that clinical benefits were maintained for at least 2 years (Licht et al., 2015).

Two further trials, one in adults and one in children, defined the proportion of patients achieving a complete TMA response as the primary endpoint, with both studies requiring the normalization of hematologic parameters sustained for ≥4 weeks as a core criterion (Fakhouri et al., 2016; Greenbaum et al., 2016). A complete TMA response was achieved in 73% of adults and 64% of children. Improvements were also observed in TMA event-free status and reduced need for TMA interventions (PE/PI sessions and/or new dialysis). eGFR improved more in children than in adults at week 26 (64 versus 29 mL/min/1.73 m^2^), and all patients discontinued PE/PI (Fakhouri et al., 2016; Greenbaum et al., 2016). These findings are corroborated by real-world evidence; for example, a Japanese post-marketing surveillance study reported TMA event-free status in 85.2% and platelet normalization in 78.3% of patients, among other outcomes (Ito et al., 2019).

From the above four clinical trials, it is evident that the interpretation of “efficacy” in aHUS is complicated by substantial disease heterogeneity, including disease stage, age, baseline renal function, genetic background, and initial treatment modalities. Consequently, no single efficacy definition applies universally across all aHUS subgroups. Table 1 outlines the primary and secondary endpoint definitions from the four pivotal trials that supported eculizumab’s approval. These definitions reflect distinct clinical priorities, ranging from hematologic normalization alone to composite endpoints combining hematologic and renal outcomes, with applicability varying according to patient characteristics and therapeutic objectives.The adult and pediatric trials (Fakhouri et al., 2016; Greenbaum et al., 2016) both adopted **c**omplete TMA response as their primary endpoint, yet a critical distinction emerged: the adult trial required renal preservation (<25% increase in serum creatinine), whereas the pediatric trial required renal improvement (≥25% decrease in serum creatinine). This discrepancy stems from children’s greater potential for recovery and the reality that many adult patients had pre-existing chronic kidney disease, making a ≥25% creatinine decrease unrealistic. Thus, the identical term “complete TMA response” carries different implications across age groups.

**TABLE 1:** Definitions of efficacy endpoints in pivotal eculizumab trials and key heterogeneity factors.

Source	Baseline population	Primary efficacy endpoints & definition	Definitions of secondary end points	Heterogeneity factors
Legendre et al. (2013)	Acute progressive TMA (n = 17)	1. Change in platelet count 2. Hematologic normalization: platelet count ≥150 × 10^9^/L and LDH ≤ upper limits of normal (ULN), confirmed by two consecutive measurements obtained at least 4 weeks	TMA event-free (≥12w), renal function, Health-related quality of life (EQ-5D)	Acute phase, 76% genetic, 100% renal impairment, 47% transplant
Legendre et al. (2013)	Chronic stable aHUS on PE/PI (n = 20)	1. TMA event-free status: required no >25% decrease in platelet count from baseline, no use of PE/PI, and no initiation of new dialysis over a minimum of 12 consecutive weeks 2. Hematologic normalization	Renal function, EQ-5D	Chronic phase, 70% genetic, 90% renal impairment, long disease duration
Fakhouri et al. (2016)	Adults with active TMA (n = 41)	Complete TMA response with renal preservation: defined as hematologic normalization together with a <25% increase in serum creatinine from baseline	Modified complete TMA response (≥25% creatinine decrease), TMA event-free status, TMA intervention rate	73% new diagnosis, 49% genetic, 59% dialysis, early treatment
Greenbaum et al. (2016)	Children (n = 22)	Complete TMA response with renal improvement: defined as achievement of hematologic normalization accompanied by a ≥25% reduction in serum creatinine from baseline	TMA event-free status, platelet normalization, eGFR improvement, CKD stage improvement, quality of life	Pediatric (5m–17y), 50% genetic, 50% dialysis, 55% no prior PE/PI

Based on this robust efficacy profile, multiple expert recommendations endorse eculizumab as first-line therapy for aHUS, replacing plasma therapy (Loirat et al., 2016; Goodship et al., 2017; Ávila et al., 2023). For confirmed aHUS, early initiation is advised because prompt complement blockade limits ongoing TMA and kidney injury (Loirat et al., 2016; Ávila et al., 2023). When a C5 inhibitor is not immediately accessible, PE should be initiated as a short-term measure (Goodship et al., 2017) and patients should be swiftly transitioned to a C5 inhibitor once it becomes accessible (Ávila et al., 2023). Initiation of eculizumab should not be delayed while awaiting complement genetic testing. Supportive care remains essential, including optimization of fluid and electrolyte balance, blood pressure control and transfusion support for anemia as needed.

## 3 Adverse events of eculizumab

In a single-arm adult trial (Fakhouri et al., 2016), all enrolled patients (n = 41) reported at least one adverse event (AE). Common AEs included headache (37%), diarrhea (32%), peripheral edema (22%), and cough (20%), among others; however, only 9% of these events were confirmed to be treatment related. Eighteen patients (44%) experienced at least one serious AE (SAE), including convulsion, pyelonephritis, dyspnea, and pulmonary edema. One such SAE, a case of meningococcal meningitis, resulted in permanent treatment discontinuation.

In a phase II clinical trial involving children (Greenbaum et al., 2016), treatment-emergent adverse events (AEs) were reported in 20 patients (91%), the majority of which were mild or moderate in severity. Common AEs included fever (50%), cough (36%), abdominal pain (32%), upper respiratory tract infection (32%), nasopharyngitis (27%), and vomiting (27%), but half of all AEs were deemed unrelated to treatment. Thirteen patients (59%) experienced serious AEs (SAEs), including fever, viral gastroenteritis, and hypertension, with no fatal events reported. A separate multicenter retrospective cohort study included 152 pediatric patients treated with eculizumab (Muff-Luett et al., 2021), which predominantly comprised patients with aHUS (47.4%), with other diagnoses including Shiga toxin-producing *Escherichia coli* HUS and unspecified thrombotic microangiopathies. Ten fatal events (6.6%) were recorded during the observation period, nine of which occurred during active therapy. Only one of these fatalities involved a patient with aHUS (1/72, 1.4%). Among the nine on-treatment deaths, the causes were cardiac arrest (n = 5), central nervous system hemorrhage (n = 3), and viral infection (n = 1). The remaining aHUS patient died several months after treatment discontinuation. Furthermore, isolated case reports indicate the necessity to monitor the potential effects of eculizumab on growth and development in pediatric and adolescent patients. Two cases involving girls aged 11 and 13 demonstrated varying degrees of bone injury during eculizumab treatment; however, whether this effect is attributable to the therapy remains under investigation (Regnier et al., 2025).

During long-term follow-up extending to 2 years (Licht et al., 2015), SAEs were reported by 78% of patients. Commonly observed AEs and SAEs included headache, accelerated hypertension, leukopenia, nausea, vomiting, and venous sclerosis at the infusion site. These symptoms occurred predominantly within the first 12 months of therapy. The frequency of AE reporting declined over time, and no new or cumulative toxicities were observed.

In clinical trials involving both adult and pediatric populations (Fakhouri et al., 2016; Greenbaum et al., 2016), increases in alanine aminotransferase (ALT) and aspartate aminotransferase (AST) levels were noted in some patients both prior to and following eculizumab administration. These transaminase elevations were not associated with concomitant clinical adverse events, and no patient discontinued treatment due to hepatotoxicity or other liver-related issues. However, a causal relationship between eculizumab and direct liver injury has not been established. One study reported hepatotoxicity in five pediatric aHUS patients following eculizumab treatment and reported that the onset of liver injury correlated with drug administration rather than the natural course of aHUS (Hayes et al., 2015). The mechanism underlying eculizumab-associated hepatotoxicity remains undefined, although a potential role for C5 in Kupffer cell and hepatocyte defense responses has been hypothesized (Schieferdecker et al., 2001). Currently, routine monitoring of liver enzymes is not standard for patients receiving eculizumab, and the true incidence of this potential adverse effect remains to be determined.

Eculizumab is administered via intravenous infusion every two to three weeks during maintenance therapy. This dosing frequency may contribute to infusion-related burdens and carry a potential risk of immunogenicity, which could manifest as allergic or hypersensitivity reactions. Nonetheless, a global safety report on eculizumab demonstrated low rates of severe infusion reactions in both adult (0.52 per 100 patient-years) and pediatric populations (0.78 per 100 patient-years) (Rondeau et al., 2019).

A significant safety concern with C5 inhibition is meningococcal infection. By inhibiting complement C5, eculizumab interrupts the terminal complement pathway and prevents the formation of the MAC, which plays a vital role in defense against *Neisseria meningitidis*. Consequently, patients receiving eculizumab are at risk of meningococcal infection that is more than 1000–2000 times greater than that of the general population (Konar and Granoff, 2017). Therefore, vaccination against the meningococcal serogroups ACWY and B is recommended before starting therapy; where possible, vaccines should be administered ≥2 weeks before the first dose (Benamu and Montoya, 2016). However, substantial risk persists even after vaccination, particularly from nongroupable (NG) strains (Nolfi-Donegan et al., 2018; Bouwman and Guchelaar, 2024). A prospective multicenter study in Turkey reported three pediatric aHUS patients who contracted NG meningococcus despite having received at least one vaccine dose (Kavaz Tufan et al., 2024). Available vaccines may lack cross-protection against NG strains, highlighting the need for additional strategies, such as long-term antibiotic prophylaxis. The protective efficacy of anti-meningococcal antibodies in the context of complement inhibition remains unclear. When eculizumab initiation coincides with vaccination, antibiotic prophylaxis is necessary. For adults, a twice-daily regimen of penicillin V 250–500 mg is recommended, with erythromycin 500 mg twice daily as an alternative for penicillin-allergic patients (Benamu and Montoya, 2016; PNH National Service, 2021). For pediatric patients, weight-adjusted dosing is recommended (Loirat et al., 2016). This prophylaxis should be continued throughout the treatment and for an additional two to three months after the cessation of eculizumab (Goodship et al., 2017; Mülling et al., 2020). Furthermore, primary vaccination with the quadrivalent meningococcal conjugate vaccine often induces a suboptimal immunogenic response, leaving approximately 80% of patients with absent or nonprotective bactericidal antibody titers. Therefore, booster doses are required to elicit an adequate immune response (Gäckler et al., 2020). In summary, patients require close clinical monitoring, proactive vaccination, and appropriate antibiotic prophylaxis during treatment, with prompt intervention and regimen adjustment upon early signs of infection.

In addition to meningococcal infection, patients treated with eculizumab also face an elevated risk for infections caused by other encapsulated bacteria, particularly *Streptococcus pneumoniae* and *Haemophilus influenzae* type b (Hib) (Campistol et al., 2015; Java et al., 2026). Although the absolute risk of pneumococcal disease is lower than that of meningococcal infection, it remains a clinically significant concern. The FDA has issued black box warnings for this medication (U.S. Food and Drug Administration, 2025). Accordingly, the US Centers for Disease Control and Prevention’s Advisory Committee on Immunization Practices (ACIP) recommends vaccination against *S. pneumoniae* and Hib prior to treatment initiation (United States Centers for Disease Control and Prevention, 2024; United States Centers for Disease Control and Prevention, 2025). For pediatric patients, these vaccinations are required as part of standard routine immunization schedules (Campistol et al., 2015; U.S. Food and Drug Administration, 2025). Additionally, C5 inhibition by eculizumab is also a recognized risk factor for invasive fungal infections, including candidiasis, mucormycosis, and cryptococcosis in rare instances (Clancy et al., 2018; Sunseri et al., 2019; Lortholary et al., 2023), possibly due to diminished cellular immune defense secondary to low C5a levels.

Overall, clinical trials have shown that eculizumab is typically well tolerated in the studied patient populations. Adequate symptomatic management of any adverse reactions is recommended.

## 4 Dosing for eculizumab

According to the prescribing information (U.S. Food and Drug Administration, 2025), the recommended eculizumab dosing regimen for aHUS is shown in Table 2.

**TABLE 2:** Prescribing information–recommended eculizumab dosing for aHUS.

Weight range	Initial phase	Maintenance phase
≥40 kg	900 mg weekly for 4 weeks	1200 mg at week 5, then 1200 mg every 2 weeks
30–40 kg	600 mg weekly for 2 weeks	900 mg at week 3, then 900 mg every 2 weeks
20–30 kg	600 mg weekly for 2 weeks	600 mg at week 3, then 600 mg every 2 weeks
10–20 kg	Single dose of 600 mg at week 1	300 mg at week 2, then 300 mg every 2 weeks
5–10 kg	Single dose of 300 mg at week 1	300 mg at week 2, then 300 mg every 3 weeks

Ter Avest et al. (2023) used nonlinear mixed-effects modeling to develop the first pharmacokinetic–pharmacodynamic (PK–PD) model of eculizumab in patients with aHUS. The model supports weight-based loading doses during induction (Table 3), PK-guided individualized dosing during maintenance and extension of the dosing interval to 4 weeks in selected patients.

**TABLE 3:** Weight-based initial loading dose of eculizumab.

Patient weight (kg)	Day 1 (induction phase), mg	Day 15 (maintenance phase), mg
≥120	2400	1200
90–<120	2100	1200
60–<90	1800	1200
40–<60	1500	1200

In the induction phase, 99.95% of patients achieved the efficacy target (classical pathway activity, CCP <10%) by day 7 with a weight-based loading dose, whereas 94.75% achieved the efficacy target with the standard regimen. This approach reduced the number of infusions and was projected to lower costs by 12.5% during the first 28 days of treatment. During maintenance, predicted target attainment was similar between the standard and individualized regimens (97.5% vs. 96.5%), whereas individualized dosing extended the interval in approximately one-third of patients and reduced drug costs. The model further suggested that 91% of patients maintain target inhibition with four weekly doses. Given the availability of the long-acting C5 inhibitor ravulizumab, the clinical need for 4-weekly eculizumab dosing may be reduced.

A subsequent prospective study by Ter Avest et al. (2024) validated the feasibility of an initial loading dose of eculizumab in adults with aHUS. In comparison to the standard regimen, the loading-dose strategy resulted in earlier effective exposure, was well tolerated (no infusion-related adverse events), reduced the number of infusions and decreased drug costs by approximately 13%.

Current Kidney Disease: Improving Global Outcomes (KDIGO) guidelines (Goodship et al., 2017) indicate that eculizumab concentrations of 50–100 μg/mL are needed to achieve complement blockade (CCP <10%) in aHUS. Fixed weight-based dosing may result in unnecessarily high drug concentrations in some patients (Gatault et al., 2015; Volokhina et al., 2015; 2017; Wehling et al., 2017). Dose optimization by extending dosing intervals or reducing doses may maintain target trough levels while reducing cost. Complement function testing may help guide dosing. Ardissino et al. (2018) adjusted regimens in patients in remission by monitoring CCP activity and TMA markers (e.g., platelet count and lactate dehydrogenase): CCP <10% allowed interval extension, >30% required shortening, and 10%–30% supported maintaining the original interval. Over a median follow-up of 26.9 months in 38 patients, no relapses were observed. Collectively, these reports indicate that extending the interval to 4–6 weeks and/or reducing the dose may be feasible in selected patients in remission, although larger prospective studies are needed for validation (Gatault et al., 2015; Volokhina et al., 2017; Wijnsma et al., 2019b; Le Tilly et al., 2024).

Eculizumab administration correlates with an extremely low incidence of anti-drug antibody development (Brodsky et al., 2008; Hanes et al., 2019). Notably, two separate studies showed that only 0%–3.1% of patients on eculizumab therapy developed anti-eculizumab antibodies, without a discernible impact on clinical response or pharmacodynamic effects of eculizumab, which continued to block complement activity (Hillmen et al., 2016). Consequently, no dose adjustments of eculizumab are needed in relation to the development of anti-drug antibodies. However, two different studies indicated that severe proteinuria is linked to initial inadequate complement inhibition (defined as CCP>10% after 7 days of eculizumab therapy) and a delayed hematological response to eculizumab therapy. Given the elevated risk of eculizumab underexposure, patients with severe proteinuria may be considered to receive an increased initial eculizumab dose (Ter Avest et al., 2023; Allinovi et al., 2024).

## 5 Treatment course of eculizumab

### 5.1 Timing of therapeutic initiation of eculizumab

When should eculizumab treatment be initiated? The earlier, the better.

Early initiation of eculizumab is strongly associated with renal recovery, improved prognosis and reduced economic burden (Legendre et al., 2013; Greenbaum et al., 2016; Matsumoto et al., 2018; Brocklebank et al., 2023; Maruyama et al., 2024; Tatematsu et al., 2024). In patients with confirmed aHUS (including recurrent disease), treatment should be initiated as early as possible, and numerous expert recommendations suggest treatment within 24 h of diagnosis. In patients with strong clinical suspicion of aHUS, early initiation is recommended, ideally within 24 h of suspicion (de Souza et al., 2023). Available evidence indicates that the presence or type of complement gene variants does not preclude response to eculizumab; therefore, genetic confirmation should not be a prerequisite for starting therapy. When access to eculizumab is limited, plasma exchange may be used as a bridging therapy until eculizumab becomes available (Ávila et al., 2023).

### 5.2 Reconsidering indefinite maintenance therapy

Eculizumab is a safe and effective first-line therapy for aHUS (Keating, 2013; Legendre et al., 2013; Greenbaum et al., 2016; de Souza et al., 2023; Bouwman and Guchelaar, 2024; Maruyama et al., 2024; Coccia et al., 2025). However, the optimal duration of treatment remains controversial. Earlier guidance favored lifelong therapy to prevent relapse, whereas emerging evidence suggests that indefinite treatment may not be required for all patients.

As mentioned above, eculizumab demonstrates an overall favorable safety profile but is still associated with inevitable adverse reactions. Although eculizumab may be economically favorable compared with plasma exchange–centered supportive care as first-line therapy for aHUS (Chen et al., 2025), the absolute cost remains substantial and can be prohibitive if lifelong treatment is assumed (Fakhouri et al., 2021a; Germeni et al., 2024). Several studies suggest that discontinuation may be feasible in selected patients, provided that relapse monitoring and prompt retreatment pathways are in place (Ardissino et al., 2015; Konar and Granoff, 2017; Merrill et al., 2017; Wijnsma et al., 2018; Chaturvedi et al., 2021; Fakhouri et al., 2021a). In a four-year prospective national multicenter study, total medical costs under an eculizumab-restricted strategy were approximately 30% of those under standard long-term dosing. Eculizumab accounts for 87% of total costs, and discontinuation of this drug does not reduce quality of life (Bouwmeester et al., 2023).

Across discontinuation studies, patients who restarted eculizumab promptly after relapse generally recovered to baseline renal function, and no serious adverse reactions were reported after reinitiation (Merrill et al., 2017; Wijnsma et al., 2018; Chaturvedi et al., 2021; Fakhouri et al., 2021a; Bouwmeester et al., 2023). Moreover, available evidence suggests that genetic background does not influence the response to retreatment (Chaturvedi et al., 2021).

In summary, eculizumab is an effective first-line treatment for aHUS; however, its long-term administration is linked to two primary concerns: increased infection risk and substantial economic burden. Available observational evidence suggests that treatment withdrawal may be feasible in selected patients, provided that close monitoring and rapid access to retreatment are ensured. On this basis, we propose that discontinuation of eculizumab may be considered in a subset of patients with aHUS.

### 5.3 Relapse after discontinuation and risk factors

The most comprehensive risk analysis of eculizumab discontinuation to date, derived from the global aHUS Registry, involved 151 patients and prospectively evaluated outcomes (including TMA relapse and renal function) following treatment cessation (Ariceta et al., 2021b). The reasons for discontinuation included physician judgment, patient preference and other factors. Overall, 22% (33/151) of the patients experienced TMA relapse, which is consistent with the findings of other studies (Fakhouri et al., 2017a; Neave et al., 2019; Fakhouri et al., 2021a). The median time to relapse was longer in adults (10.2 months) than in children (5.1 months), supporting closer early monitoring in pediatric patients.

This study further indicates that TMA relapse is not associated with the duration of continuous therapy; sustained disease stability induced by prolonged eculizumab administration does not assure a diminished risk of TMA relapse after discontinuation. However, these findings diverge from those reported by Neave et al. (2019), who retrospectively assessed outcomes in 22 patients with acute aHUS following eculizumab discontinuation. Their analysis suggested that an abbreviated initial treatment duration was correlated with elevated relapse risk, suggesting that at least 6 months of eculizumab therapy should be given prior to discontinuation. Methodological disparities may explain the discrepancy between these studies. Nonetheless, both investigations, along with additional research, support the association between genetic mutations and elevated relapse rates (Fakhouri et al., 2017a; Schaefer et al., 2018).

The presence and type of complement gene variants do not affect the response to eculizumab in aHUS patients (Fakhouri et al., 2017a); however, individuals with pathogenic variants in complement-related genes experience more frequent relapses than those without such mutations. In several reported studies on eculizumab discontinuation in aHUS patients, no relapses were observed in patients without complement gene mutations (Ardissino et al., 2015; Fakhouri et al., 2017a; Menne et al., 2019; Fakhouri et al., 2021a; Bouwmeester et al., 2023). In studies investigating the association between pathogenic complement gene variants and the relapse risk following eculizumab discontinuation (Fakhouri et al., 2017a), patients with CFH gene variants presented the highest relapse rate (72%), followed by those with MCP variants (50%). However, relapses in MCP variant carriers often occur later after eculizumab discontinuation. Furthermore, studies indicate that pediatric aHUS patients with MCP mutations have more favorable long-term renal outcomes than adults do (Fremeaux-Bacchi et al., 2013); nevertheless, their higher relapse risk warrants attention. Klämbt et al. (2021) demonstrated via Kaplan-Meier analysis that pediatric aHUS patients with MCP mutations experienced relapse rates of 7%, 29%, and 40% at 1, 3, and 5 years, respectively, without long-term maintenance therapy, representing an annual relapse risk of approximately 10%.

In a prospective, multicenter, open-label trial (NCT02574403) conducted by Fakhouri et al. (2021a), the overall relapse rate of aHUS patients after treatment discontinuation was 23% (13/55). Among the recurrent cases, 92% (12/13) carried complement gene variants (patients with CFH or MCP variants exhibited the highest relapse rate of 50%), and no difference in relapse risk was observed between children and adults. Female sex, the presence of complement gene variants, and elevated sC5b-9 levels at the time of drug discontinuation were significantly associated with relapse risk.

Ariceta et al. (2021b) compared extrarenal manifestations in patients with and without relapse after eculizumab discontinuation. In addition to pathogenic complement variants and a family history of aHUS, a history of extrarenal involvement before eculizumab initiation independently increased relapse risk. Patients with previous extrarenal manifestations exhibited higher relapse rates (69.7% vs. 51.3%) and a greater incidence of extrarenal events following discontinuation (63.6% vs. 16.8%). Gastrointestinal involvement was most common, followed by central nervous system and cardiovascular manifestations. The authors therefore suggested continuing treatment in patients with a history of extrarenal manifestations; KDIGO guidance similarly advises against discontinuation when extrarenal disease persists (Goodship et al., 2017).

In summary, the main factors contributing to relapse after eculizumab discontinuation are pathogenic complement gene mutations (notably CFH and MCP variants), a history of extrarenal manifestations, and a greater tendency for earlier relapse in pediatric patients. Although children with MCP mutations have better long-term renal outcomes, they still face a persistently high relapse risk. Biomarkers, specifically elevated sC5b-9 levels at the time of discontinuation, are significantly correlated with relapse. Despite the ongoing controversy regarding the relationship between treatment duration and relapse, the high-risk status of genetic mutations has been consistently established.

### 5.4 Discontinuation timing

Current evidence indicates that planned discontinuation of eculizumab may be feasible in selected patients with aHUS. Discontinuation strategies informed by complement genetics are considered both reasonable and safe (Chaturvedi et al., 2021; Fakhouri et al., 2021a). The decision to stop eculizumab treatment mainly hinges on evaluating post-discontinuation relapse risk. However, multiple other factors also need to be taken into account. These factors include the patient’s age, the extent of renal function recovery (partial or complete), whether aHUS affects the native or transplanted kidney, the presence of severe extrarenal manifestations, and the patient’s personal preference for discontinuation. In regard to discontinuation, crucial factors include the availability of genetic testing, patient compliance with monitoring, and support from healthcare coverage (Germeni et al., 2024).

Currently, studies continue to explore the optimal treatment duration, and viewpoints on the timing of discontinuation differ (Supplementary Table S2).

In the above studies, most analyses have indicated that complement gene variants are risk factors for aHUS relapse after eculizumab discontinuation. Moreover, even though the data from Chaturvedi et al. (2021) showed no statistically significant correlation between rare complement gene mutations and aHUS relapse risk, the post-discontinuation relapse rate was 40% in patients with CFH or MCP mutations and 33.3% in those with complement mutations other than CFH and MCP. Thus, complement gene variants can still be regarded as risk factors for aHUS relapse. Notably, this study indicated that non-adherence was associated with a greater risk of relapse compared to patients with complement gene variants and was linked to the only case of aHUS relapse resulting in renal deterioration and death within the study cohort. Therefore, discontinuation of eculizumab should be approached with particular caution in patients with complement pathogenic variants and poor adherence.

We gathered and analyzed relevant research data on eculizumab discontinuation in aHUS patients (Supplementary Table S3). Current real-world evidence suggests that disease relapse following eculizumab cessation is not rare, with relapse rates reported to vary widely across studies from approximately 0%–50%. Overall, about one-quarter of patients experience disease relapse after discontinuation. Relapse events predominantly manifest in the early post-discontinuation period, with multiple cohort studies consistently showing that the initial 3–6 months following cessation constitute a high-risk interval for relapse and some studies reporting a median time to relapse as brief as 2–5 months, which underscores the critical importance of vigilant monitoring and close follow-up in the immediate post-discontinuation phase. Moreover, the risk of relapse postcessation varies significantly depending on patients' genetic backgrounds, with those harboring pathogenic variants in complement pathway genes (such as CFH and MCP) having a significantly higher relapse rate after discontinuation, suggesting that genetic risk stratification holds substantial clinical value in discontinuation decision-making. Importantly, existing data have originated from mainly observational or retrospective studies, which present considerable heterogeneity in baseline patient characteristics, duration of eculizumab therapy, discontinuation criteria, and follow-up protocols; thus, these findings are more applicable for risk assessment rather than supporting a standardized discontinuation strategy. Overall, these data endorse exploring the feasibility of discontinuation with careful patient selection, while highlighting the need for intensive early monitoring post-discontinuation and the establishment of rapid retreatment protocols.

**FIGURE 1:**
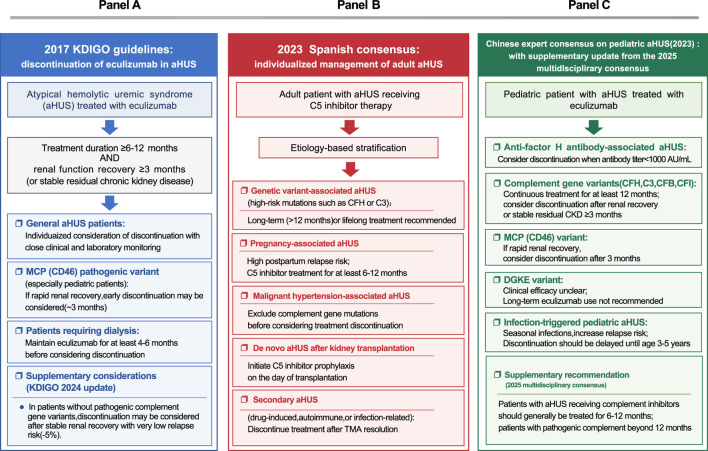
Summary of guideline- and consensus-based recommendations on treatment duration and withdrawal of terminal complement inhibition in patients with aHUS. Panel **(A)** summarizes the 2017 KDIGO guideline recommendations on the discontinuation of eculizumab (Goodship et al., 2017), with supplementary considerations derived from the 2024 KDIGO conference discussions (Vivarelli et al., 2024). Panel **(B)** illustrates the 2023 Spanish expert consensus for adult aHUS (Ávila et al., 2023). Panel **(C)** displays recommendations from the 2023 Chinese expert consensus on pediatric aHUS (Pediatric aHUS Professional Committee of China Alliance of Rare Diseases et al., 2023), alongside supplementary considerations from the 2025 multidisciplinary expert consensus (Multidisciplinary Consensus Collaboration Group on Atypical Hemolytic Uremic Syndrome, 2025). These recommendations are shown as summaries of published guidance and do not replace individualized clinical decision-making.

Although current expert consensus documents offer guidance on eculizumab use in aHUS (Goodship et al., 2017; Pediatric aHUS Professional Committee of China Alliance of Rare Diseases et al., 2023; Ávila et al., 2023; Vivarelli et al., 2024; Multidisciplinary Consensus Collaboration Group on Atypical Hemolytic Uremic Syndrome, 2025), there is no standardized recommendation regarding the timing and strategy of treatment discontinuation (Figure 1). On the basis of available evidence and clinical experience, a stratified discontinuation strategy is recommended, incorporating both biological risk factors and patient-related considerations.

Discontinuation may be considered in patients who meet one or more of the following criteria: (i) severe infection or hypersensitivity requiring treatment cessation; (ii) absence of pathogenic complement gene variants (or presence of benign MCP variants only), along with complete renal recovery or stable kidney function for ≥3 months; and (iii) absence of severe extrarenal manifestations.

Discontinuation should be avoided or strongly discouraged in patients with any of the following features: (i) pathogenic variants in CFH, C3, CFB or CFI, CFH gene rearrangements, or anti-CFH antibodies; (ii) variant-associated high-risk polymorphisms; (iii) history of disease relapse or a positive family history; (iv) kidney transplant recipients; and (v) severe extrarenal manifestations.

### 5.5 Monitoring after discontinuation of eculizumab

In recent years, personalized management of eculizumab treatment regimens has become a critical advancement in aHUS therapy. An increasing amount of successful clinical practice offers evidence that backs the shift from fixed-dosing schedules to dynamic, personalized treatment approaches for patients (Mikačić and Marić, 2024). In this context, rigorous relapse monitoring is a crucial element of eculizumab discontinuation strategies.

The 2023 Spanish consensus on adult aHUS recommends regular monitoring, including hematological parameters, urinalysis, blood pressure and symptoms/signs of infection, after eculizumab discontinuation (Ávila et al., 2023). The suggested schedule is weekly in the first month, monthly from months 2 to 6, and then every 2 months thereafter.

Currently, serum complement markers (e.g., C3, C4, C5b-9, AP50, or CH50) have yet to be established as reliable biomarkers for evaluating disease activity or predicting relapse in patients with aHUS, and their value in posttreatment monitoring remains restricted. As reported by Noris et al. (2014), the core pathological mechanism of aHUS is localized complement overactivation in endothelial cells, and there are no marked differences in serum C3 levels or plasma C5a/SC5b-9 levels between the acute and remission phases of the disease. *In vitro* experiments demonstrated that serum from patients with aHUS leads to significant C3 and C5b-9 deposition on the surface of unstimulated or ADP-activated human microvascular endothelial cells, whereas serum from healthy controls does not have this effect. On the basis of these findings, this study is the first to suggest that serum-induced endothelial C5b-9 deposition could be a sensitive means to guide eculizumab dose adjustment and dosing intervals.

Further research has shown that, compared with serum testing alone, exposing endothelial cells to the plasma of aHUS patients and considering the interaction between the complement and coagulation cascades can improve the sensitivity and stability of C5b-9 deposition detection (Palomo et al., 2019). Therefore, the detection of endothelial C5b-9 deposition is considered to have potential value in evaluating the disease activity of aHUS and guiding individualized treatment. In particular, it may contribute to the adjustment of the eculizumab dosage and the assessment of relapse risk (Galbusera et al., 2019). Additionally, Stevens et al. (2023) developed an in vitro model using conditionally immortalized glomerular microvascular endothelial cells, which can assess the abnormal activation of the complement system on the glomerular microvascular endothelium, thereby simulating the process of complement-mediated endothelial injury. This model holds potential for the evaluation of individualized treatment strategies for aHUS.

These endothelial function–related assays remain largely research tools and are not yet part of routine clinical practice. The value of C5b-9 for post-treatment monitoring requires further validation, and the applicability of C5b-9 assays in kidney transplant–associated aHUS may also be limited (Duineveld et al., 2024).

## 6 Use of eculizumab in special forms of aHUS

### 6.1 Kidney transplant-associated atypical hemolytic uremic syndrome

Kidney transplant–associated aHUS can be classified into two categories. First, in patients whose native kidneys fail due to aHUS (end-stage kidney disease, ESKD), the risk of relapse after transplantation is high: posttransplant recurrence occurs in approximately 60% of patients, and up to 91.6% of recurrences result in graft failure (Bresin et al., 2006). Second, *de novo* aHUS can occur after transplantation in patients without prior aHUS. Although *de novo* cases account for approximately 90% of all post-transplant microangiopathies documented in the literature (Reynolds et al., 1998), these largely encompass a spectrum of complement dysregulation triggered by transplant-specific factors.

For kidney transplant recipients with ESKD secondary to aHUS, mutation-based risk stratification is essential for guiding post-transplant management decisions. In accordance with the KDIGO guidelines (Goodship et al., 2017), relapse risk is categorized into three tiers, with intermediate risk including no mutation identified, isolated CFI mutations, complement gene variants of unknown significance, and persistent low-titer FHAA, and low risk including isolated MCP mutations and persistently negative FHAA. Prophylactic eculizumab is recommended for high- and intermediate-risk patients, whereas low-risk individuals require no therapeutic intervention. Emerging evidence suggests that this stratification can be refined on the basis of the presence or absence of a homozygous high-risk CFH haplotype (CFH-H3; tgtgt), where patients lacking pathogenic variants in circulating complement regulators (CFH, CFI, C3/CFB) and devoid of the double copy of the high-risk CFH haplotype exhibit minimal relapse rates, potentially obviating the need for eculizumab prophylaxis (Zuber et al., 2019).

A personalized eculizumab-based prevention strategy, guided by risk stratification, significantly reduces the risk of aHUS relapse, particularly in high-risk patients (Zuber et al., 2019). Furthermore, it notably decreases the likelihood of dialysis and improves graft function (Siedlecki et al., 2018). The recovery of the graft subsequent to relapse may be associated with the timing of eculizumab administration after relapse. Initiating treatment within 1 week of aHUS relapse leads to more favorable outcomes than delayed treatment does (Zuber et al., 2012; 2019).

A meta-analysis revealed that the prophylactic use of eculizumab can reduce the relapse rate of aHUS to 6.3% and lower the graft loss rate due to TMA to 5.5%. In contrast, when the drug is used therapeutically, the graft loss rate due to TMA reaches 22.5% (Gonzalez Suarez et al., 2019). A comparative study of eculizumab prophylaxis versus rescue therapy in kidney transplant recipients (Duineveld et al., 2025), showed that the cohort receiving prophylactic eculizumab experienced a marked decrease in aHUS relapse. However, the study evaluated the lifelong use of prophylactic eculizumab, while rescue resulted in a significant reduction in the total eculizumab dosage. Furthermore, in specific patient populations, rescue therapy achieves graft survival rates on par with those of prophylactic therapy while notably reducing drug costs, which supports the implementation of personalized treatment strategies.

The evidence on the discontinuation of prophylactic eculizumab after kidney transplantation is limited (Table 4). The available studies suggest that discontinuation may be feasible in selected patients. Notably, the two reported cases of relapse in Levi et al. (2017), Zuber et al. (2019) occurred in high-risk patients, implying that discontinuation may be more reasonable in intermediate-risk patients, although this requires confirmation in larger studies. The optimal duration of prophylaxis and the best time to stop therapy remain uncertain.

**TABLE 4:** Discontinuation of prophylactic eculizumab after kidney transplantation.

Source	Prophylaxis cohort (n)	Relapses (n)	Follow-up (months)	Risk category
Ardissino et al. (2014)	1	0	14.2	High risk
Fakhouri et al. (2021a)	3	0	5.4-24^a^	Intermediate; Low risk
Portoles et al. (2021)	2	0	1-12.03	Intermediate risk
Matar et al. (2014)	3	0	21-26	High; Low risk
Levi et al. (2017)	2	1	1-9	High; low risk
Zuber et al. (2019)	12	1	56.6 (0.03–108)^a^	High; Intermediate risk

Follow-up: period after discontinuation of prophylactic eculizumab, reported as a range unless otherwise specified.

^a^ Follow-up duration refers to overall study follow-up rather than follow-up after prophylactic eculizumab discontinuation.

### 6.2 Pregnancy-associated atypical hemolytic uremic syndrome

Pregnancy-associated aHUS (P-aHUS) is a clinically important subtype that requires balancing maternal–fetal safety with effective disease control. Eculizumab, a monoclonal antibody that targets complement C5, is currently considered a first-line therapeutic option for P-aHUS.

Registry data suggest that pregnancy-triggered aHUS shares similar clinical characteristics, genetic backgrounds and treatment responses with other forms of aHUS, supporting complement-targeted therapy for P-aHUS (Fakhouri et al., 2021b). Eculizumab treatment is associated with reduced risks of chronic kidney disease and ESKD and with fewer maternal and fetal complications (Fakhouri et al., 2021b; Meena et al., 2025). Existing evidence suggests a low risk during lactation; eculizumab does not appear to accumulate in fetal plasma, and *in utero* exposure has not been shown to affect neonatal complement function, suggesting low teratogenic potential (Hallstensen et al., 2015). These findings are indirectly supported by clinical experience in pregnant patients with paroxysmal nocturnal hemoglobinuria (Kelly et al., 2015; Servais et al., 2016). Eculizumab should be initiated promptly once P-aHUS is confirmed. Dose and/or dosing frequency may need adjustment as pregnancy progresses and the volume of distribution increases, guided by individualized monitoring (Servais et al., 2016; Lim et al., 2023).

In addition to kidney transplantation-associated and P-aHUS, which are the focus of this review, other special subtypes exist in clinical practice, including malignant hypertension (MHT)-associated aHUS, metabolism-related aHUS, infection-induced aHUS, as well as cancers and medications (e.g., calcineurin inhibitors commonly used in renal transplantation, and chemotherapeutic agents such as gemcitabine) as secondary causes. Owing to their extremely low incidence and marked phenotypic heterogeneity, further elucidation of their pathological mechanisms and treatment strategies requires larger-scale cohort studies.

## 7 From eculizumab to ravulizumab

Ravulizumab, a long-acting C5 inhibitor derived from eculizumab, is approved for aHUS (European Medicines Agency, 2019; Syed, 2021; U.S. Food and Drug Administration, 2022). The introduction of a histidine substitution in the complementarity-determining region increases its dissociation rate from C5 under acidic conditions (pH 6.0). Concomitantly, two point mutations within the Fc region increase its binding affinity for the human neonatal Fc receptor (FcRn) (Sheridan et al., 2018). These synergistic modifications substantially extend the terminal half-life of ravulizumab from approximately 11 days–51.8 days (Sheridan et al., 2018), allowing maintenance dosing every 4–8 weeks depending on body weight (Syed, 2021).

The efficacy of ravulizumab was established in two phase III, single-arm, multicenter trials with a duration of 26 weeks involving distinct age cohorts (Rondeau et al., 2020; Ariceta et al., 2021a). At 26 weeks, a complete TMA response was achieved in 53.6% of adults and 78% of pediatric patients, with improvements in platelet count, LDH and kidney function (Rondeau et al., 2020; Ariceta et al., 2021a). Direct comparative trials versus eculizumab are lacking. Indirect comparisons and meta-analyses typically indicate comparable efficacy and safety; however, these findings should be interpreted cautiously because of differences in study populations, endpoint definitions and follow-up. A meta-analysis incorporating both clinical trial and real-world data indicated no significant difference in efficacy between the two C5 inhibitors (Bernuy-Guevara et al., 2020). Another indirect comparison (Tomazos et al., 2022) revealed numerical differences in outcomes (e.g., the proportion of patients requiring dialysis or mortality events) between the eculizumab and ravulizumab study cohorts. In the absence of adjusted comparative data, these differences cannot be attributed to the therapeutic agents themselves and should not be regarded as evidence of superior or inferior efficacy or safety. Consequently, the choice between therapies in practice should be guided by practical considerations such as dosing frequency, cost, and availability.

In the current management of aHUS, eculizumab and ravulizumab exhibit comparable safety and efficacy profiles. However, ravulizumab is often favored because of its lower economic burden and reduced dosing frequency (Shahid and Qayyum, 2023). A web-based survey (Mauch et al., 2023) revealed that the vast majority of adult patients (94.0%) and all caregivers of pediatric patients expressed a preference for ravulizumab over eculizumab, with infusion frequency cited as a primary factor in treatment selection. Levy et al. (2022) compared the treatment time, productivity loss and total cost between the two agents for aHUS. Their analysis revealed that, compared with 10 mg/mL eculizumab, 10 mg/mL and 100 mg/mL ravulizumab reduced the total treatment time by 44%–52% and 69%–74%, respectively, corresponding to productivity loss savings of 56% and 73%, respectively. Compared with eculizumab, ravulizumab was associated with an approximately 32%–40% reduction in total treatment cost, with cost savings being more substantial in pediatric patients.

Considering the advantages of enhanced quality of life and lower treatment costs, patients requiring long-term therapy often transition from eculizumab to ravulizumab (Schönfelder et al., 2024). The real-world efficacy and safety of this transition have been substantiated by multiple studies. A multicenter retrospective analysis involving adults demonstrated that among 32 HUS patients (including 10 kidney transplant recipients) who switched after at least 3 months of eculizumab therapy, no new TMA events or deterioration in renal function occurred during up to 12 months of follow-up on ravulizumab (Schönfelder et al., 2024). The incidence and profile of adverse events were similar to those associated with eculizumab, with the majority of adverse events assessed as not related to the complement inhibitor (Schönfelder et al., 2024). In a clinical trial of pediatric patients converting from chronic eculizumab to ravulizumab treatment, renal and hematological parameters remained stable after the switch, with no unexpected safety signals identified (Tanaka et al., 2021). Additionally, research involving kidney transplant recipients with aHUS indicates that the transition from eculizumab to ravulizumab is well tolerated, maintaining graft function without introducing new treatment-related safety concerns (Busutti et al., 2024; Gaeckler et al., 2025).

In conclusion, ravulizumab has a similar mechanism of action and comparable efficacy to that of eculizumab while offering an approximately fourfold longer half-life. Ravulizumab offers significant benefits, such as lower treatment costs and less frequent dosing, positioning it as a key therapeutic alternative for patients with aHUS. Currently, eculizumab is still more commonly used in clinical practice, likely owing to its longer market presence, greater physician familiarity, clinical experience, and extensive evidence base. Nevertheless, with the continued accumulation of real-world evidence and experience, ravulizumab has emerged as an important treatment option.

## 8 Discussion

Eculizumab is currently established as a first-line therapy for aHUS, with multiple clinical studies demonstrating its clear benefits in improving hematologic outcomes and promoting renal function recovery. The available evidence consistently indicates that early initiation of eculizumab therapy is closely associated with better renal recovery and improved overall prognosis.

The long-term management of aHUS patients on eculizumab therapy should focus on individualized treatment strategies. Personalized approaches, such as weight-based loading protocols or extended dosing intervals, may maintain adequate complement inhibition and clinical efficacy while reducing the risks associated with excessive complement blockade and reducing the long-term economic burden. In addition, eculizumab discontinuation may be cautiously considered in carefully selected patients, with the recognition that relapse after treatment withdrawal is not uncommon and may be influenced by multiple factors, including pathogenic complement variants and prior extrarenal manifestations. Consequently, close follow-up and early relapse monitoring after discontinuation are essential to ensure safe treatment withdrawal. When relapse occurs, prompt reinitiation of eculizumab therapy generally enables recovery of renal function to baseline or near-baseline levels. However, several limitations should be acknowledged. Most existing studies on drug discontinuation are observational in design, with potential confounding from age heterogeneity. In addition, the definition of relapse and monitoring frequency vary considerably across studies. Furthermore, publication bias may exist, as cases with successful drug withdrawal are more likely to be reported. These factors collectively limit the generalizability of our findings.

For specific aHUS subtypes, tailored management strategies are needed. For example, in transplant-associated aHUS, genetic information may facilitate relapse risk stratification and guide the development of more precise preventive and therapeutic approaches. Treatment strategies for pregnancy-associated aHUS should account for physiological changes during pregnancy, requiring flexible eculizumab dosing adjustments to ensure both maternal and fetal safety while preserving therapeutic efficacy.

Notably, the emergence of the long-acting C5 inhibitor ravulizumab has provided a new therapeutic option for aHUS. Compared with eculizumab, its reduced dosing frequency may lessen the treatment burden during long-term therapy and has the potential to play an increasingly important role in future aHUS management strategies.
