# Supplementary material for: Therapeutic advances in eculizumab for atypical hemolytic uremic syndrome: a narrative review
Source: Front Pharmacol. 2026 May 28;17:1834141. doi: 10.3389/fphar.2026.1834141 (PMC13253509; doi:10.3389/fphar.2026.1834141)
Supplement: Supplementary file 1 [file DataSheet1.docx]

**Supplementary Material**

**Supplementary Table S1. Key efficacy outcomes of eculizumab in aHUS pivotal trials.**

|  | **Trial 1 (Legendre et al., 2013)** | **Trial 2 (Legendre et al., 2013)** | **Trial 3 (Fakhouri et al., 2016)** | **Trial 4 (Greenbaum et al., 2016)** | **Key Notes** |
| --- | --- | --- | --- | --- | --- |
| Study population (n) | Adults/Adolescents (n = 17) | Adults/Adolescents (n = 20) | Adults (n = 41) | Pediatric Patients (n=22) |  |
| Median treatment duration | 26 weeks | 26 weeks | 26 weeks | 26 weeks |  |
| Normalization of platelet count - no./total. (%) | 14/17 (82) | 18/20 (90) | 40/41 (98) | 21/22 (95) |  |
| Mean increase in the platelet count (× 10^9^/L) | 73 | NA | 135 | 164 | Greater increase observed in pediatric patients and those treated earlier. |
| Lactate dehydrogenase ≤ upper limit of normal range - no./total no. (%) | 14/17 (82) | 19/20 (95) | 37/41 (90) | 18/22 (82) | LDH ≤ upper limit of normal indicates resolution of hemolysis. |
| Hematologic normalization (%) | 76 | 90 | 88 | 82 |  |
| Mean increase in estimated glomerular filtration rate (mL/min/1.73 m^2^) | 32 | 6 | 29 | 64 | Early treatment initiation correlates with greater renal recovery. |
| TMA event-free status (%) | 88 | 80 | 90 | 95 |  |
| Complete TMA response (Renal Improvement) (%) | 65 | 25 | 56 | 64 |  |
| Complete TMA response (Renal Preservation) (%) | NA | NA | 73 | NA |  |
| Discontinuation of Dialysis - no./total no. (%) | 4/5 (80%) | NA | 15/19 (79%) | 9/11 (82%) | Data reflect patients who were on dialysis at baseline and subsequently discontinued during the study period. |
| Discontinuation of PE/PI (%) | 88 | 100 | 100 | 100 | Proportion of patients who stopped plasma exchange/infusion during eculizumab therapy. |
| Meningococcal Infections (n) | 0 | 0 | 2 | 0 | Both cases occurred despite vaccination; underscores need for prophylaxis and vigilance. |
| Death (n) | 0 | 0 | 0 | 0 |  |

The specific criteria for "Complete TMA response", "TMA event-free status", and "Hematologic normalization" vary based on clinical heterogeneity and are extensively defined in Table 1 in the main text.

**Supplementary Table S2. Recommended eculizumab regimens for aHUS management**

| **Source** | **Relapse risk factors** | **Study parameters** | **Recommendation** |
| --- | --- | --- | --- |
| Ariceta (Ariceta et al., 2021) | History of extrarenal manifestations, pathogenic variants, or a family history of aHUS | Among 151 eculizumab-responsive aHUS patients, extrarenal manifestations prior to treatment: 69.7% (relapse) vs 51.3% (non-relapse); pathogenic variants: 30.3% (relapse) vs 15.0% (non-relapse); CFH/MCP variants: 18.2%/15.0% (relapse) vs 10.4%/9.6% (non-relapse). | Patients with impaired renal function, family history, pathogenic variants, extrarenal manifestations, or a history of transplantation should not discontinue treatment. Those with an initial renal response to eculizumab have a lower risk of eGFR decline upon relapse. Treatment should be promptly reinitiated upon suspicion of relapse. |
| Ardissino (Ardissino et al., 2015) | pathogenic variants | In 16 aHUS patients post-eculizumab discontinuation: 5 relapsed and 11 had continuous remission. 4 of the 5 relapsed patients had CFH-related aHUS. | Avoid treatment discontinuation in kidney transplant recipients, carry CFH mutations, or have an eGFR <20 mL/min/1.73 m². Discontinuation may be considered in patients with FHAA only if antibody titers are <2.5 times the upper limit of normal. |
| Fakhouri (Fakhouri et al., 2017) | pathogenic variants | Among 38 eculizumab-discontinued patients (9 children, 29 adults): 12 (31%) relapsed. Among 11 patients with CFH mutation, 8 (72%) relapsed. Among 8 patients with MCP mutation, 4 (50%) relapsed. No relapse in 16 patients without rare complement variants detected. | Patients without complement gene mutations may consider discontinuing treatment after 6-12 months; decisions for CFH/MCP mutation carriers require careful consideration; for FHAA-positive patients, discontinuation may be considered after immunosuppressive therapy if antibody levels fall below 1000 AU/ml. |
| Coccia (Coccia et al., 2025) | pathogenic variants | Four pediatric FH antibody-mediated HUS patients received eculizumab first-line. Immunosuppression followed by antibody confirmation. At 6 months, all achieved significant anti-FH titer reduction, permitting eculizumab discontinuation. One patient with a persistent titer >2000 AU/ml received single-dose rituximab. No relapses or treatment-related complications occurred during follow-up after eculizumab withdrawal. | A 6-month C5 inhibitor course may reverse TMA and improve renal function in severe anti-FH antibody-mediated HUS. Combining immunosuppressants (e.g., MMF) reduces antibody titers, shortens treatment duration, and lowers relapse risk. Discontinuation is feasible when anti-FH titers fall below 1000 IU/ml. |
| Chaturvedi (Chaturvedi et al., 2021) | Non-adherence | 25 non-transplant acute aHUS patients discontinued eculizumab: 18 per protocol, 7 due to non-adherence. Overall relapse: 20% (5/25). Non-adherence group relapse: 42.8% (3/7), protocol group: 11.1% (2/18). Among relapses, 4 achieved remission; 1 died from recurrent aHUS and non-adherence-related hypertensive emergency. | Eculizumab may be discontinued if:  (1) No active TMA manifestations;(2) Renal function normal/stabilized (≥3 months); (3) No identifiable triggers;(4) No prior transplant for aHUS;(5) Demonstrated adherence to monitoring. |

**Supplementary Table S3. Studies evaluating eculizumab discontinuation in patients with aHUS**

| **Source** | **Total N** | **Age, years** | **Eculizumab exposure before discontinuation, months** | **Follow-up after discontinuation, months** | **Discontinued, n** | **Recurrence after discontinuation, n/N (%)** | | | **Time to recurrence after discontinuation, median**  **(months)** |
| --- | --- | --- | --- | --- | --- | --- | --- | --- | --- |
|  |  |  |  |  |  | **Overall** | **MCP** | **CFH** |  |
| Ariceta (Ariceta et al., 2021) | 151 | NA | 11.5 (IQR 4.8–19.2) | 27.6 (IQR 16.8–40.8) | 151 | 33/151 (22%) | NA | NA | 5.0 overall (5.1 children; 10.2 adults) |
| Fakhouri (Fakhouri et al., 2017) | 108 | 30 (range 2–79) | 17.5 (range 2–50) | 22 (range 5–23) | 38 | 12/38 (31%) | 8/11(72%) | 4/8 (50%) | NA |
| Ardissino (Ardissino et al., 2015) | 16 | NA | 4.3 (range 0.5–14.4) | 21 (range 0.4–40.0) | 16 | 5/16 (31%) | 0/2 | 3/3 (100%) | ≤6 |
| Coccia (Coccia et al., 2025) | 4 | 6–11 (range) | 6 (range 4–15) | 12 (range 8–24) | 4 | 0 | NA | NA | NA |
| Chaturvedi (Chaturvedi et al., 2021) | 31 | NA | 2.37 (IQR 1.06–9.70) | ≥3^a^ | 25 | 5/25 (20%) | NA | NA | NA |
| Fakhouri (Fakhouri et al., 2021) | 55 | NA | 16.5 (mean; range 0.95–59) | 19.8 (range 5.4–24) | 55 | 13/55 (23%) | 6/12(50%) | 3/6 (50%) | 10.2 (range 1.6–22.1) |
| Ardissino (Ardissino et al., 2024) | 244 | 30.6 (IQR 10.1–43.9) | 6.9 (IQR 1.2–16.6) | 68.4 (range 30–105.6) | 81 | 23/81 (28.4%) | NA | NA | 4.8 (range 1.2–15.6) |
| Baskin (Baskin et al., 2022) | 63 | 4.0 (IQR 2.7–10.2) | 16 ± 7 (mean ± SD) | 50.4 (IQR 26.4–84) | 18 | 4/18 (22.2%) | NA | NA | ≤6 |
| Ito S (Ito et al., 2022) | 40 | <18 | 15.23 (median) | NA | 18 | 2/18 (11%) | NA | NA | NA |
| Menne (Menne et al., 2019) | 93 | NA | 19.6 (range 0.2–86.9) | 31.2 (range 0.7–95.1) | 42 | 21/42 (50%)^b^ | NA | NA | ≤30 |
| Merrill (Merrill et al., 2017) | 17 | 46 (range 19–69) | 3.0 (range 0.5–18.2) | 10.2 (range 1.2–46.3) | 15 | 3/15 (20%) | NA | NA | 2.0 (range 1.8–3.3) |
| Wijnsma  (Wijnsma et al., 2018) | 20 | 24 (IQR 7–40) | 3.8 (range 1.3–14.7) | Adults: 23.6; children: 27.5 (median) | 17 | 5/17 (29%) | NA | 4/8 (50%) | 7.5 (range 1–12) |
| Ardissino  (Ardissino et al., 2018) | 38 | 25 (range 0.5–60) | 2.6 (range 0.4–24.6) | 26.9 (range 0.8–80.9) | 9 | 0 | NA | NA | NA |

Values are reported as medians (IQRs or ranges) unless otherwise stated. Recurrence refers to TMA recurrence (aHUS relapse). “≤X” indicates that recurrence occurred within X months after discontinuation.

^a^ Minimum follow-up after discontinuation.

^b^ Reinitiation of eculizumab was used as a surrogate for off-treatment TMA recurrence; reinitiation may also occur for transplant preparation or other clinical considerations.

Abbreviations: MCP, membrane cofactor protein (CD46); CFH, complement factor H; IQR, interquartile range; NA, not available; TMA, thrombotic microangiopathy.

**Reference**

Ardissino, G., Cresseri, D., Mancuso, M. C., Capone, V., Porcaro, L., Amico, V., et al. (2024). Outcome of atypical hemolytic uremic syndrome: role of triggers and complement abnormalities in the response to C5 inhibition. *J. Nephrol.* 37, 1017–1026. doi: 10.1007/s40620-023-01873-9

Ardissino, G., Possenti, I., Tel, F., Testa, S., Salardi, S., and Ladisa, V. (2015). Discontinuation of Eculizumab Treatment in Atypical Hemolytic Uremic Syndrome: An Update. *Am. J. Kidney Dis.* 66, 172–173. doi: 10.1053/j.ajkd.2015.04.010

Ardissino, G., Tel, F., Sgarbanti, M., Cresseri, D., Giussani, A., Griffini, S., et al. (2018). Complement functional tests for monitoring eculizumab treatment in patients with atypical hemolytic uremic syndrome: an update. *Pediatr. Nephrol.* 33, 457–461. doi: 10.1007/s00467-017-3813-2

Ariceta, G., Fakhouri, F., Sartz, L., Miller, B., Nikolaou, V., Cohen, D., et al. (2021). Eculizumab discontinuation in atypical haemolytic uraemic syndrome: TMA recurrence risk and renal outcomes. *Clin. Kidney J.* 14, 2075–2084. doi: 10.1093/ckj/sfab005

Ariceta, G., Fakhouri, F., Sartz, L., Miller, B., Nikolaou, V., Cohen, D., et al. (2021). Eculizumab discontinuation in atypical haemolytic uraemic syndrome: TMA recurrence risk and renal outcomes. *Clin. Kidney J.* 14, 2075–2084. doi: 10.1093/ckj/sfab005

Baskin, E., Fidan, K., Gulhan, B., Gulleroglu, K., Canpolat, N., Yilmaz, A., et al. (2022). Eculizumab treatment and discontinuation in pediatric patients with atypical hemolytic uremic syndrome: a multicentric retrospective study. *J. Nephrol.* 35, 1213–1222. doi: 10.1007/s40620-021-01212-w

Chaturvedi, S., Dhaliwal, N., Hussain, S., Dane, K., Upreti, H., Braunstein, E. M., et al. (2021). Outcomes of a clinician-directed protocol for discontinuation of complement inhibition therapy in atypical hemolytic uremic syndrome. *Blood Adv.* 5, 1504–1512. doi: 10.1182/bloodadvances.2020003175

Coccia, P. A., Alconcher, L. F., Ferraris, V., Lucarelli, L. I., Grillo, M. A., Arias, M. A., et al. (2025). Eculizumab as first-line treatment for patients with severe presentation of complement factor H antibody-mediated hemolytic uremic syndrome. *Pediatr. Nephrol. Berl. Ger.* 40, 1041–1047. doi: 10.1007/s00467-024-06530-2

Fakhouri, F., Fila, M., Hummel, A., Ribes, D., Sellier-Leclerc, A.-L., Ville, S., et al. (2021). Eculizumab discontinuation in children and adults with atypical hemolytic-uremic syndrome: a prospective multicenter study. *Blood* 137, 2438–2449. doi: 10.1182/blood.2020009280

Fakhouri, F., Fila, M., Provôt, F., Delmas, Y., Barbet, C., Châtelet, V., et al. (2017). Pathogenic Variants in Complement Genes and Risk of Atypical Hemolytic Uremic Syndrome Relapse after Eculizumab Discontinuation. *Clin. J. Am. Soc. Nephrol.* 12, 50–59. doi: 10.2215/cjn.06440616

Ito, S., Hataya, H., Ashida, A., Hamada, R., Ishikawa, T., Ishikawa, Y., et al. (2022). Eculizumab for paediatric patients with atypical haemolytic uraemic syndrome: full dataset analysis of post-marketing surveillance in Japan. *Nephrol. Dial. Transplant.* 38, 414–424. doi: 10.1093/ndt/gfac150

Menne, J., Delmas, Y., Fakhouri, F., Licht, C., Lommelé, Å., Minetti, E. E., et al. (2019). Outcomes in patients with atypical hemolytic uremic syndrome treated with eculizumab in a long-term observational study. *BMC Nephrol.* 20, 125. doi: 10.1186/s12882-019-1314-1

Merrill, S. A., Brittingham, Z. D., Yuan, X., Moliterno, A. R., Sperati, C. J., and Brodsky, R. A. (2017). Eculizumab cessation in atypical hemolytic uremic syndrome. *Blood* 130, 368–372. doi: 10.1182/blood-2017-02-770214

Wijnsma, K. L., Duineveld, C., Volokhina, E. B., Van Den Heuvel, L. P., Van De Kar, N. C. A. J., and Wetzels, J. F. M. (2018). Safety and effectiveness of restrictive eculizumab treatment in atypical haemolytic uremic syndrome. *Nephrol. Dial. Transplant.* 33, 635–645. doi: 10.1093/ndt/gfx196
